# Unexpectedly high prevalence of asthenopia in Australian school children identified by the CISS survey tool

**DOI:** 10.1186/s12886-020-01642-3

**Published:** 2020-10-12

**Authors:** Barbara M. Junghans, Serap Azizoglu, Sheila G. Crewther

**Affiliations:** 1 School of Psychology and Public Health, La Trobe University, Bundoora, Victoria 3086 Australia; 2 School of Optometry and Vision Science, University of New South Wales Sydney, Sydney, NSW 2052 Australia; 3 School of Medicine, Deakin University, Geelong, Victoria 3220 Australia

**Keywords:** Asthenopia, Middle school; CISS questionnaire, Refractive error

## Abstract

**Background:**

To date there have been few systematic attempts to establish the general prevalence of asthenopia in unselected populations of school-aged children. Thus, the aim of this study was to determine whether the incorporation of Borsting et al’s 2003 Revised Convergence-Insufficiency Symptom Survey (CISS) into a general school vision screening could aid in the identification of children with visual discomfort and indicate the need for further investigation.

**Methods:**

Vision screening of an unselected middle school population investigated and analysed the incidence of self-reported nearwork-related visual discomfort via the CISS along with distance and near visual acuities plus non-cycloplegic autorefraction using a Shin-Nippon NVision-K 5001.

**Results:**

Of the 384 unselected students approached in Grades 6–9, 353 participated (92.2%, mean 13.2 ± 1.4 years). The mean CISS score for the population without amblyopia and/or strabismus (96.0% of all students) was 16.8 ± 0.6, i.e., 45% of students in this cohort had CISS scores greater than one standard deviation above the mean found by Borsting et al. in 2003 during their validation study of the CISS on 9 to 18 year old children without binocular anomalies. Regression analyses indicated significantly higher (*p* < 0.001) mean CISS scores for the 3.2% who were hyperopes ≥ + 2.00D by non-cycloplegic autorefraction (27.7 ± 14.7) and for those who were amblyopic (24.3 ± 6.6) or strabismic (34.0 ± 9.8). The mean CISS score of 31.6 ± 9.0 for non-amblyopic/strabismic students having near vision poorer than 0.1 LogMAR was significantly higher (*p* < 0.001) than for those with good acuity.

**Conclusion:**

The most important finding of this study was the high incidence of asthenopia in an unselected population and that refractive status per se was not a major contributor to CISS scores. The results highlight the usefulness of the CISS questionnaire for assessment of visual discomfort in school vision screenings and the need for future exploration of near binocular vision status as a potential driver of asthenopia in school students, especially given current trends for frequent daily use of computers and handheld devices and necessarily prolonged accommodative-convergence effort at near, both at school and at home.

## Background

Anecdotal reports regarding the existence of ocular discomfort or asthenopia (e.g. eyestrain, nearwork-associated headaches, blurred or double vision and a range of ocular sensations) have been in existence since the Middle Ages (see [1, 2]). In recent times there has been an upsurge of scientific interest in asthenopia, largely in adults (see overviews [3, 4]), with the increasing prevalence of computers and hand-held devices [5–11] and 3-D movies [12–15]. By contrast, a systematic meta-analysis of the prevalence of asthenopia in paediatric populations up to age 18 years by Vilela [16] recently noted the relative scarcity of well-designed studies. The few systematic attempts to establish the general prevalence of asthenopia in normal unselected populations of school children [17–20] reflect a prevalence ranging from 15 to 32% of non-clinical patients [21] possibly due to the differences in the definition of oculo-visual discomfort, or the criteria for classification of symptoms, and the differing conditions under which the data was collected (e.g. age, socioeconomic grouping, time of day when tested).

Traditionally ophthalmic clinicians have considered asthenopic symptoms in children and young adults to relate to refractive error (both low and high degrees of hyperopia [2, 22–24], and to some extent hyperopic astigmatism [22, 25–28]), given the accommodation/convergence triad and evidence that uncorrected hyperopia is often associated with excessive accommodative demand [29]. Dysfunctional accommodation and convergence may also induce ocular discomfort during prolonged periods of reading or attending to computers or handheld devices at near as is now the accepted norm for most young adolescent-aged individuals. Such prolonged nearwork both at school and at home has also been independently reported to be associated with an increased prevalence of asthenopic symptoms [19, 30–34], that include headaches, psychological and head/neck muscular strain [22, 35, 36].

To elicit asthenopic impact qualitatively and quantitatively, questionnaires are increasingly being utilized as survey instruments (e.g. [3, 17–19, 28, 37, 38]). Five studies that meet meta-analysis inclusion criteria [18–20, 30, 39] all reported a relatively high overall prevalence of asthenopia (ranging from 12.4 to 32.2%) using survey instruments. Thus, we aimed to use the Convergence Insufficiency Symptom Survey (CISS) [40, 41], that has been validated for detection of CI in children and young adults, as a tool to investigate the prevalence of asthenopic symptoms during a general screening of unselected middle school students (i.e. a cohort of students where no selection process was used other than grade at school and parental and student consent to participate) attending a metropolitan school. Although the CISS survey was initially designed to quantify symptoms in convergence insufficiency (CI) patients and monitor treatment outcomes, it has been used by several other groups ‘off label’, so to speak, across a range of investigations into visual symptomatology driven by accommodative dysfunction or behavioural or learning disorders [4, 42, 43]. Indeed, the CISS shares many questions pertaining to aspects of oculo-visual discomfort in common with the psychological tool created by Conlon et al. [38] to explore and quantify psychophysiological visual stress responses to spatial patterns similar to those encountered during reading. The reliability of the CISS tool in children has previously been established [41].

We chose to include all potential subjects regardless of visual status, despite the original studies involving the use of the CISS having excluded patients with amblyopia and/or strabismus [40, 41]. One might intuitively sense that amblyopes and strabismics would report a higher frequency and intensity of asthenopic symptoms, and therefore would bias the mean outcomes of school CISS screenings. However, the CISS profile for amblyopes and strabismics has not hitherto been established or controlled for, or compared to other refractive error status groups. Furthermore, to explore potential clinical associations with asthenopia, we chose to use habitual refractive status (rather than cycloplegic refraction which removes the stress from the prolonged compensatory accommodative-convergence response) along with the screening variables visual alignment and visual acuity in an Australian unselected non-clinical population. Ophthalmic testing such as subjective refraction and extensive investigation of binocular vision status was excluded in order to determine whether the variables open to screening by a layperson in an educational setting could be indicative of the requirement for further assessment. It is important to acquire an early understanding of manageable factors in the aetiology of a child’s asthenopic symptoms engendered by our modern lifestyle [44], so as to minimise the adverse impact of asthenopia upon the individual’s education [37, 45–52].

## Methods

Students were recruited for a visual screening from a private school of predominantly second generation Australian-born Lebanese ethnicity [53] after obtaining permission from the Principal to send letters to parents. All students in grades 6 to 9 (aged 10–15 years) were invited to participate in a vision screening. Three hundred and fifty-four students out of the possible 384 students (92.2%) returned informed consent forms signed by a parent or guardian. No other selection process was used. Absence from school on the day of testing accounted for the majority of the missing students. Testing was carried out during normal school hours. All procedures were approved by the Human Ethics Committee of La Trobe University, Melbourne, Australia, and conform to the Declaration of Helsinki of 1975 (as revised in Tokyo in 2004). Percentile scores of non-verbal intelligence established using the Raven’s Coloured Progressive Matrices Test (1998 Edition) were available and indicated the expected range of intelligence (i.e. in terms of mental age) for a non-selective general middle school.

Prior to any visual assessments, the revised CISS [41] (see Table 1) was administered to all students by the class teacher during a school period. Instructions were to “carefully read each question and tick one of the five possible answers”. Scoring of the CISS as per Table 1 ranges from zero (no symptoms) to 60 (highly symptomatic) and was undertaken independently and prior to visual acuity and refraction testing. CISS scores were not available to those undertaking clinical assessments.

**Table 1:**
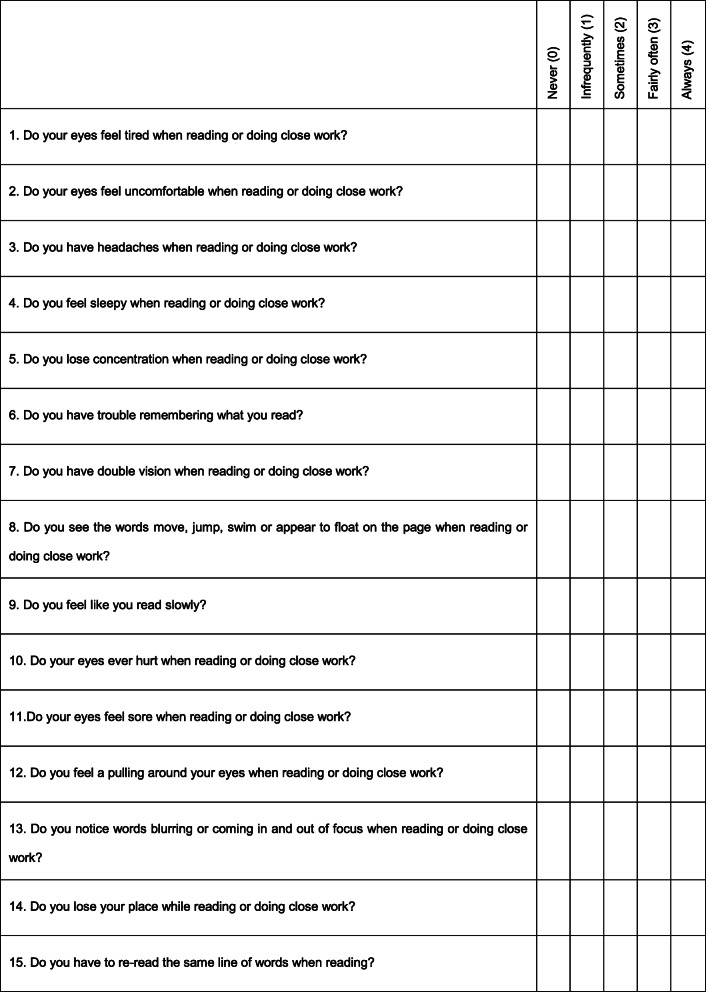
The CISS Questionnaire [41]

The vision screening began with questions regarding (i) a history of previous eye examinations, (ii) experience of spectacle wear and (iii) experience with patching. Students were then examined in their habitual refractive state or with spectacles if available, as this reflects how the majority operate day-to-day. Visual acuity was tested monocularly and binocularly at distance using an internally illuminated distance ‘ETDRS’ (Early Treatment Diabetic Retinopathy Study) LogMAR chart (Precision Vision, La Salle, USA) and at near using the ‘ETDRS’ card at a measured 40 cm wearing their correction if available, followed by non-cycloplegic autorefraction, and lastly, a cover test to identify strabismus. Amblyopia was defined as a difference in visual acuity between the two eyes of at least 2 lines, or, a bilateral reduction in expected acuity of at least two lines for both distance and near acuities, with an associated strabismic eye movement during the cover test.

Given that our interest is asthenopia in an unselected school population doing their normal daily school tasks, we chose to use non-cycloplegic autorefraction that was undertaken using a Shin-Nippon NVision-K 5001 autorefractor (Grand-Seiko Company, Fukijama, Japan) - an open-field machine that provides a wide and distant view, and so minimizes stimulation of ‘proximal’ accommodation when compared with closed-box autorefractors [54, 55]. This design choice was supported by the studies of Chat and Edwards [56] and Choong et al. [54] using an earlier model open-field Grand Seiko (WR-5100 K), that indicated that in children 3 years younger than the current study, non-cycloplegic autorefraction has clinically acceptable accuracy (i.e. within 0.13D) and repeatability against the binocular endpoint of non-cycloplegic subjective refraction (sensitivity 0.91, specificity 0.97). Accuracy and vertex distance settings on the instrument were set to 0.12 D and 12 mm respectively. Ten sequential autorefraction readings were made in rapid succession and simultaneously downloaded to a custom-designed Labview program (Labview, National Instruments, Austin, TX). The means were converted to spherical equivalent refraction (SER) for analysis. A dioptric cut-off for myopia of ≤ − 0.50D SER was superimposed with a criterion that unaided vision should be LogMAR 0.2 (6/9.5) or poorer to exclude the possibility of instrument myopia due to involuntary accommodation effects from an awareness of nearness [57]. In line with clinical decision-making relating to autorefractions, astigmatism ≤0.50 DC was disregarded (the cut-off so chosen as the Shin Nippon Autorefractor is considered to only be accurate to ±0.50Dcyl in 96% of measures) [55].

The initial classification of refraction was based on the Refractive Error Study in Children (RESC) definitions [58], that is, clinical myopia if the SER was equal to or below − 0.50DS and hyperopia if equal to or above + 2.00DS, with emmetropia thus defined as SER between − 0.50D and + 2.00D and astigmatism defined as cylinder findings ≥0.75 DC. The degree and axis of astigmatism was only considered in cases where the cylindrical autorefractions were found to be over 0.50 DC, and axes were then subdivided into with-the-rule/against-the-rule/oblique axis presentations. Anisometropia was deemed to exist if a SER difference between right and left eyes was greater than 1.00D.

Students were referred to the family eye care practitioner for reassessment and/or monitoring if it was found they had refractive error as per the RESC definitions, poor visual acuity or moderate to high CISS scores.

### Data analysis

As SER readings for right and left eyes of non-amblyopes/non-strabismics were highly correlated (*r* = .92, *p* < .0001), right eye results alone are presented. The data from students with amblyopia or strabismus (*n* = 14) were not included in the general CISS score analysis relating to refractive error as refractions between the two eyes are usually anisometropic and there is no a priori expectation of a high correlation between the two eyes [59]. However, the data from these students were examined in relation to their CISS scores per se and are reported as a separate group. One student with a refractive error of − 16.00DS in both eyes was excluded from all data analysis.

In deference to what constitutes the most appropriate CISS cut-off scores to denote significant asthenopia for the current study, the validation studies on children aged 9–18 years by Borsting et al’s [41] and Rouse et al’s [50] Convergence Insufficiency Treatment Trial (CITT) have been used as a reference base (notably, the visual status of the student was masked in the latter but not the former). Borsting et al’s first study [41] yielded a mean CISS score of 8.4 ± 6.4SD (range 2 to 15) in 9 to 18 year olds in a clinical population diagnosed as having no binocular vision anomalies, whereas those diagnosed with CI yielded a mean score of 30.8 ± 8.4 (range of 14 to 50). Based on a categorization of with/without CI against the CISS score, a cutoff of CISS≥16 yielded a sensitivity of 95.7% and specificity of 87.5% in this first study, a cutoff which also happened to be equivalent to the ‘the mean CISS score plus one standard deviation’. The group’s later masked study [50] yielded mean CISS scores of 10.4 ± 8.1, which were not significantly different to those in the earlier study, and lead these researchers to declare that the cut-off to separate children with an unusual degree of symptomatology from those with a normal degree should remain at ≥16.

Therefore, we arbitrarily chose to use the following criteria (i) that scores 0–15 be regarded as ‘normal’ (i.e. using the CITT mean + one standard deviation (=14.8 [41] to define the upper boundary of normality), and (ii) scores of 16–30 be designated ‘moderately symptomatic’ (i.e. using the CITT mean + 1 to 3.5 standard deviations [41]), and (iii) scores of 31–60 be deemed ‘highly symptomatic’ (i.e. using > 3.5 standard deviations above the CITT mean [41]).

Analyses of differences in CISS scores between the RESC-defined refractive groups were evaluated using a one-way independent ANOVA (despite quite different group sample sizes). Post-hoc comparisons were made using the Scheffe Method. Similar analyses were carried out with further subdivision of the refractive categories into milder hyperopia ≥ + 0.75D but <+ 2.00D and mild myopia of ≤ − 0.50D but > − 2.00D. Mean values for age, SER and CISS are quoted with the standard deviation (SD). Comparison of CISS scores between refractive groups is facilitated on the graphs by displaying 95% confidence intervals. The CISS scores were examined by regression analyses to determine the contribution of poor visual acuity at distance or near to the CISS score.

## Results

A total of 354 out of the 384 enrolled students (92.2%) completed the CISS survey and were screened for refractive status, distance and near visual acuity, and ocular alignment via cover test. Fifty-four percent were female. The mean age was 13.2 ± 1.5 years (range 10 to 15 years). Fourteen students (4.0%) were found to have manifest misaligned visual axes (strabismus) and/or amblyopia. Mean distance acuity for students without strabismus and/or amblyopia was LogMAR -0.04 ± 0.14 (6/6^+ 2^) and for near LogMAR -0.03 ± 0.08 (6/6^+ 1^). Thirteen students (3.6%) had distance visual acuity poorer than LogMAR 0.1 (6/7.5) in both eyes.

**Fig. 1:**
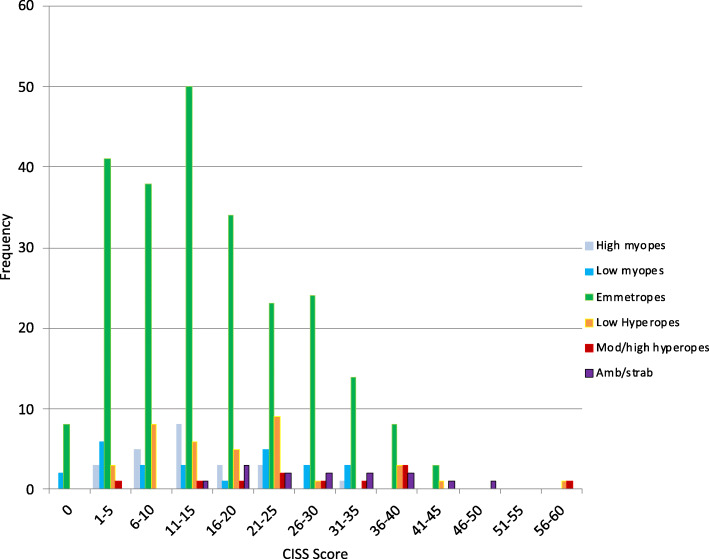
Histogram illustrating the frequency of total CISS survey scores for 353 students according to refractive category and the presence or not of amblyopia/strasbismus

The frequency distribution for CISS scores across all participants (*n* = 353) is shown in Fig. 1. The mean CISS score for all participants was 16.8 ± 11.1 (range 0 to 56). Age was not found to be a significant factor in CISS scores (*r* = 0.019; *p* = 0.722).

The mean spherical equivalent refraction for the 339 non-amblyopic and/or non-strabismic students was + 0.05 ± 1.33D (range − 7.77 to + 3.54D). The majority of these 339 students were emmetropic according to the RESC definition of > − 0.50DS to <+ 2.00DS (*n* = 278, 82.0%) while according to the autorefractor there were significantly smaller proportions with myopia (*n* = 50 or 14.8%) and hyperopia (*n* = 11 or 3.2%). Five students (10% of myopic autorefractions) appeared to be pseudo-myopic as their autorefraction SERs were more myopic than − 1.00DS but their unaided distance visual acuity was in the range LogMAR − 0.1 to 0.1 (6/4.8 to 6/7.5) leaving only 13.3% of students as diagnostically myopic. A further three students with autorefractions indicating myopia were deemed by their unaided acuities to have myopia of lesser degree than measured by the autorefractor. Of students without amblyopia or strabismus, 275 had astigmatism as detected by the autorefractor (range 0.12 to 3.21 DC), with seventy-six (22.4%) having astigmatism > 0.50 DCyl. Twenty-six students were anisometropic (mean anisometropia of 2.17 ± 0.99DS), of whom 7 were also amblyopic/strabismic. Fifty-three of the 339 students (15.6%) had previously been prescribed spectacles.

For students who had been prescribed spectacles, the mean myopic autorefractor finding with a spherical component ≤ − 0.50DS was − 3.06DS ± 1.85DS, and the mean hyperopic finding with a spherical component ≥ + 0.50DS was + 1.45DS ± 0.73DS. Unfortunately, it was not recorded whether a student actually had their spectacles with them. However, from the correlation of spectacle ownership and autorefractor findings with visual acuity, it is assumed that a majority of myopes would have been wearing their spectacles at school, although this cannot be estimated for the hyperopes as none were greater than + 2.75D SER.

### Amblyopic/strabismic students

These students were not included in the particular refractive groups as the amblyopic eye will be more hyperopic [59]. For the 14 students operationally-defined as having amblyopia and/or strabismus, the mean CISS of 28.9 ± 9.8 was highly significantly higher (*p* < 0.001) than the mean CISS scores for those having neither of these conditions (Figs. 1 and 3). The 7 students with amblyopia without strabismus represented just 1.98% of all 353 students and returned the highest symptom scores (mean 33.4 ± 10.1, range 20 to 47). Importantly, 4 students among this 7 were unable to recall having had an eye examination previously despite a mean symptom score of 36.5. Another seven students (2.0% of all students) were found to have strabismus and returned moderately high near symptom scores (24.4 ± 7.7, range 15 to 37). There was no significant difference regarding the degree of reported near symptoms between strabismic students with or without acuity loss.

### Non-amblyopic/non-strabismic students

**Fig. 2:**
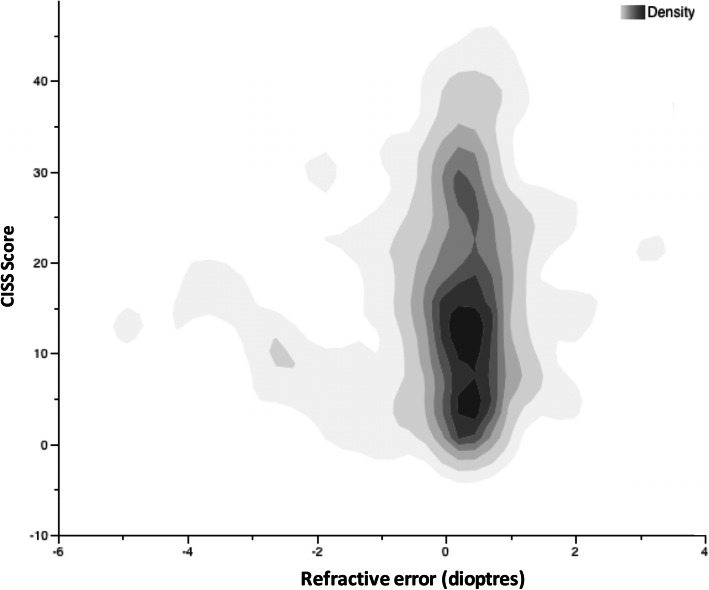
A heatmap illustrating CISS score density versus spherical equivalent refractive error for students without obvious binocular impairment

The mean symptom survey score for students without obvious binocular impairment was 16.3 ± 10.9 and the median was 15. Figure 2 shows the individual CISS scores versus SER status as a heat map. Of these students, 33.9% scored 16 to 30 (i.e. were moderately symptomatic) and the remaining 11.5% scored over 30 (highly symptomatic). CISS scores were significantly higher (*p* < 0.001) for students with near visual acuity that was poorer than 0.1 LogMAR (CISS mean 31.6 ± 9.0) than those with better acuity (CISS mean 15.7 ± 10.6). Near acuity contributed to 5.4% of the variance of the CISS scores (R^2^ = 0.05435); however, distance acuity was not a factor.

**Fig. 3:**
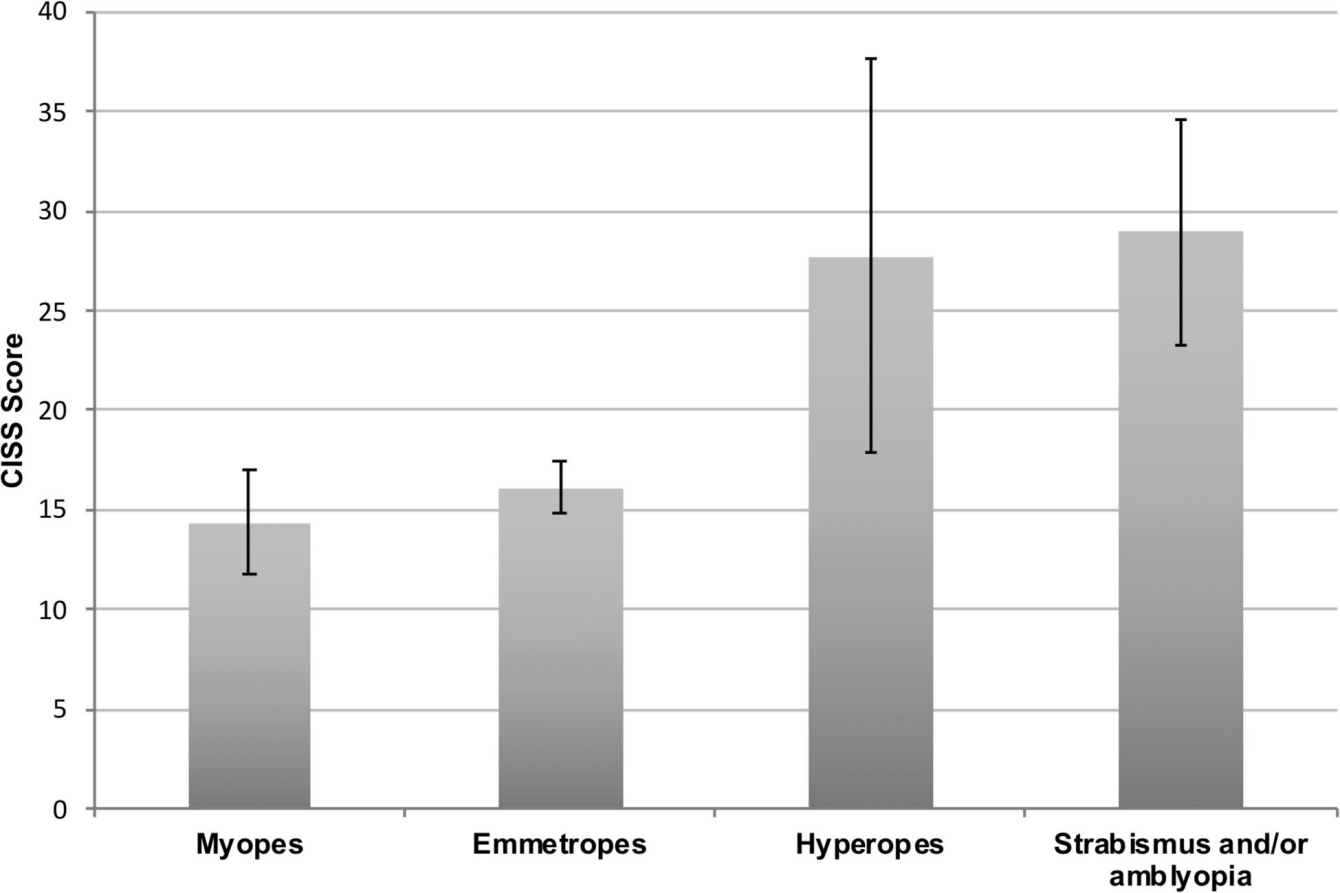
Mean (±95% confidence interval) CISS scores for students without amblyopia or strabismus according to category of spherical equivalent refraction (using the RESC classification [58]), and for those with either amblyopia or strabismus or both

CISS scores according to refractive error groupings are shown in Table 2. Overall there was little correlation between refractive error as a continuous variable and CISS scores (correlation coefficient = 0.13). However, a significant effect was found when refraction was considered instead in major refractive groups (i.e. the emmetropes and mild hyperopes are combined as per RESC guidelines as shown in Fig. 3) (*F*(4, 334) = 4.09, *p* = .003).

**Table 2:** Mean CISS scores (± 95% confidence intervals) in children without amblyopia and/or strabismus according to their refractive category and for those with amblyopia and/or strabismus

	Spherical equivalent refraction	N	Mean CISS	95%CI
Strabismus and/or amblyopia	Any refractive error	14	28.9	5.1 (23.8, 34.0)
No strabismus and/or amblyopia	Myopia : SER ≤-2.00DS	24	15.0	4.3 (10.7, 19.3)
	Myopia: SER ≤-0.50DS and >-2.00DS	26	15.1	4.2 (10.9, 19.3)
	Emmetropia: SER >-0.50DS and <+0.75DS	237	16.1	1.3 (14.8, 17.4)
	Hyperopia: SER ≥+0.75 DS and <+2.00DS	41	17.3	3.4 (13.9, 20.7)
	Hyperopia: SER ≥+2.00DS	11	27.7	9.9 (17.8, 37.6)
	Astigmatism >0.50DC	76	16.6	2.3 (14.3, 18.9)
	Astigmatism 0.25 to 0.50DC	158	17.2	1.9 (15.3, 19.1)

The degree of astigmatism (as found during distance viewing through the autorefractor) did not affect the frequency of near symptoms (see Table 2). CISS scores were unrelated to the presentation of the axis of astigmatism (with-the-rule, against-the-rule or oblique). CISS scores were also unrelated to type of astigmatism (i.e. myopic, hyperopic or mixed), the latter two of which will potentially incur an accommodative demand if improved focus is sought by the subject. Of note is the fact that no students with mixed astigmatism (that is, with focal lines straddling the retina) had been prescribed spectacles.

The percentage of students previously prescribed spectacles was 83% of those with myopia ≤ − 2.00, 31% for those with lower myopia ≤ − 0.50, 7% for those approximately emmetropic, 15% for those with low hyperopia <+ 2.00, and 18% for moderate to high hyperopes. Post-hoc tests showed no significant difference in CISS scores between students with or without a history of previously prescribed spectacles in the various refractive groups.

## Discussion

From an evidence-based practice viewpoint, the most important finding of this study is that self-reported asthenopia of moderate extent using the CITT Study Group’s criteria [41, 50] appears to be extremely common (45%) in Australian middle school children. As expected, students with obvious binocular impairment reported the highest number of symptoms, followed closely by those with significant hyperopia (≥ + 2.00D). However, more surprisingly, the RESC-defined ‘emmetropic‘group (refractive errors up to + 2.00DS) demonstrated a mean symptom survey score of approximately 16, that is, a score one standard deviation above that found for those without strabismus and having normal binocular vision in the CITT clinical population using the same survey tool [41]. Notably, myopes reported only marginally less severe symptomatology than did emmetropes. On the other hand, despite previous suggestions that astigmatism, particularly hyperopic astigmatism, might be a driver of asthenopia [25–28], we found no systematic variation relating to astigmatic imagery. Thus, all refractive groups reported a comparatively high prevalence of symptoms on the CISS survey that was normed on individuals without binocular problems, suggesting that moderate to high refractive error alone cannot remain the sole criterion for managing visually related issues in children. Rather, as we [60] have previously noted that 39.2% of young elementary school children (aged 8.3 ± 2.4 years) showed binocular vision anomalies, binocular vision assessment should be considered a necessary addition to standard vision screenings.

### Prevalence of symptoms

The number of students in our unselected non-clinical population who reported symptoms is particularly high compared with the pooled prevalence of 19.7% experiencing asthenopia sourced from the five studies considered by Vilela et al. [16] in their meta-analysis (one study from Australia in 2006, two from Sweden in 2006 and 2007, and two from India in 2011 and 2013) but using different survey tools. Against studies using the same CISS tool, the frequency of symptoms experienced by our students (score of 16.3 ± 10.9) is on average nearly twice that found in the CITT studies [41] on children without binocular anomalies (CISS 8.1 ± 6.2), and is also higher than the scores found by Marran [42] on students without hyperopic or astigmatic refractive error or reduced visual acuity(CISS 10.3 ± 8.2).

The most likely differences in prevalence and frequency of symptoms may be, in part, due to sampling differences with respect to individual difference among the population, age range, and the protocols used. In particular, most previous uses of this tool have used clinical populations and either excluded those with binocular vision anomalies in the control group [41, 50] or excluded only those with amblyopia and strabismus [19], or had no exclusion criteria for the control groups [17, 18, 20]. A further population difference for the CITT studies is Borsting et al’s exclusion of children with attention deficit hyperactivity disorder (ADHD) or learning difficulties [40, 41]. As it is our intention to develop a protocol that uses simple tools during an in-school vision screening in order to identify those students reporting asthenopic symptoms and hence require referral for further, more intensive clinical examination, for whatever reason, we deliberately chose to have no exclusion criteria. Furthermore, as the school involved in our study enrols children typical of mainstream schools in Australia, at this stage of developing our protocol we had no reason to separate out students known to have ADHD or learning difficulties. Pertinent to this, Barnhardt et al. [61] found children with parent-reported ADHD did score higher on the CISS (22.2 ± 11.0 versus 13.8 ± 9.2), particularly on the ‘performance’ questions but not the ‘ocular’ questions. The consequence of our study having some students with ADHD or learning difficulties would be that our mean CISS score would be expected to be higher. Notably however, any causal relationships between increased CISS scoring on performance-related questions, binocular dysfunction and academic aspects such as poor reading, overall academic performance and the existence of ADHD have not been established [43]. The reliability of the CISS itself should also be considered when comparing studies. Borsting et al. [41] carried out an appraisal across 2 weeks of the reliability of the CISS on students aged 9 to 18 years who had CI, and found the test to be reliable with the difference in CISS scores being 0.98 ± 5.7.

In general, increasing symptom prevalence has been found with increasing age [61, 62]. Rouse et al. [62] found an increase in CISS scores of 2.6 when comparing the scores of adults to children, presumably due to greater periods of prolonged near work for adults [61] and increased duration of handheld electronic devices for leisure [44]. Our students were on average nearly 2 years older (13.17 ± 1.4 years) than those in the CITT group’s [41] earlier study (mean 11.4 ± 2.2 years), and this age difference may account for a portion of the increase in symptoms found in our students. In a later study, the CITT group had a larger group of children, and found an age effect existed with increasing CISS scores in older students, which has been corroborated by others [61]. Similarly, the children in Marron’s sample were on average 2 years younger than our students and exhibited a lower mean CISS [42]. An Australian study by Ip et al. [18] undertaken on children just 6 years old, far younger than those of the current study, revealed a low prevalence of symptoms (12.4%). In Sweden, Abdi et al. [17] found that 26.7% of children in grades 4 and 8 in Sweden (aged 10 to 16 years) reported near symptoms. In the study by Tiwari in India [20], the children were 2 years younger than our children and 32.2% reported asthenopic symptoms.

A further contributor to sampling differences could be the differing distribution of refractive errors across participants in each study in the meta analyses. We and others [30] found symptoms to be greater in hyperopes, albeit, our numbers with moderate to high hyperopia were relatively small. The relative distributions of refractive errors for both CITT studies [41, 50] was reported in their later study and indicated that respectively 38.9/30.4% were myopic > − 0.50DS (twice as many as the current study), 3.7/0.0% were hyperopic > + 1.00DS (compared with 10.3% in the current study) and 57.4/69.6% were emmetropic. The Swedish study [17] and Australian study by Ip et al. [18] used reasonably representative whole-of-school sampling. On the other hand, the Indian study [20] examined children who until recently had been child-workers in the gem industry but were placed into further education as a result of government initiatives, with children from a similar socioeconomic background used as controls.

Further biases may come from the years between data collection, as recent evidence from studies in adults suggests that the rapid increase in the use of computers and handheld devices has led to an increase in symptomology [44, 63]. It is pertinent to note that the data from the current study was collected at a time not dissimilar to the other studies already mentioned. Another bias that has been raised, concerns the ethnicity of the students in the current study. We have found no evidence in the literature linking racial or ethnic differences and the prevalence of asthenopia. Our students are mainly second-generation Australians living a typical western lifestyle and being schooled in English according to a government mandated curriculum. Currently there is only one study from the Middle East [64]. This study used the CISS to monitor asthenopia in university students undergoing treatment for CI and found at the end of treatment a very significant improvement in CISS scores to values of 13.3 ± 7.5 and 11.3 ± 4.5 depending on which CI treatment. These post-treatment scores are well within the range of ‘normal’ CISS scores as determined by our Australian-born students of Lebanese background.

### Symptom survey value

The use of formal surveys of symptomatology to replace less standardized structured history-based studies to determine potential existence of asthenopia, particularly relating to refractive error, has been limited [17, 19, 40–42, 45, 65–69]. The advantage of a survey such as the CISS, is that the frequency with which symptoms occur is also established. A well-designed survey will usually ask important questions in more than one way as a check on reliability. Surveys also offer the advantage of the participant being left alone to consider and re-consider answers without dealing with the social stress of communicating with an unknown adult. Additionally, surveys can at times be administered by a lay person. The CISS was originally administered orally to each subject [40, 41, 50] whereas in our case the teacher administered the CISS to the whole class as a written exercise. It is unknown whether this change in protocol for the CISS has yielded different scores compared with one-on-one administration. The student’s capacity to read and understand could be brought into question, although in the current case the students were aged 10 to 15 years, schooling was in English, plus, their scores from the Raven’s Coloured Progressive Matrices Test (1998 Edition) and teacher assessment would suggest < 1% potentially did not have the cognitive capacity to read and understand the questions. However, a potential disadvantage of a survey is that it is not possible to ask follow-up questions if an answer sounds unlikely [70]. For example, how does the child conceptualize the time frame relating to each question: was it whilst they were actually answering the question, or, in recent times, or as far back as they could recall? For students who own spectacles, did they differentiate between comfort with/without spectacles? Clearly with respect to spectacle wear, if spectacles have been appropriately prescribed and are regularly worn, then the overall CISS scores should be lower. These possible differences in temporal-precision underpinning a student’s interpretation of symptoms have not been explored. Data regarding compliance and associated CISS scores for spectacle wear by students in the current cohort was not available. Hence, the finding here that high CISS scores remain common is all the more perturbing and demands follow-up using compliance diaries.

The potential value of a written survey of symptoms such as the CISS questionnaire is highlighted by the high scores we have attained from the amblyopes/strabismics and higher hyperopes. Although originally designed for use in cases of dysfunctional convergence, claims have been made that the CISS primarily identifies the binocular anomaly of accommodative insufficiency more so than convergence insufficiency [42]. Thus, we see the CISS as potentially useful in informing not only the debate regarding identification of binocular anomalies, but also the debate regarding criteria for prescribing for children and identifying tolerance of uncorrected hyperopia. Our high scores argue for the use of the CISS as an evidence base in clinical decision-making relating to prolonged near work issues in children [23, 42, 71–77].

Despite the greater degrees of asthenopia reported by individuals with strabismus and/or amblyopia, it could be argued that the CISS may not be necessary for children with ocular misalignment or poor visual acuity as they would be detected during an oculo-visual screening. However, four of the seven amblyopic students with reduced acuity without conspicuous strabismus could not recall a previous visual exam despite a visual acuity difference between their two eyes of between 2 and 5 lines. This finding of a high mean CISS for these students argues for the validity of including the CISS in a battery of tests conducted by laymen (e.g. by school teachers) to identify students visually at risk and worthy of further detailed follow-up. One could also argue that perhaps the high CISS scores from this category of students contributed significantly to our choice of the term ‘unexpectedly high’ in the title of this article. However, the mean CISS for non-amblyopic/strabismic students (16.3 ± 10.9) was only 0.5 lower than the mean CISS including amblyopic/strabismic students (largely due to the relatively low numbers of students with these particular visual problems). Thus, the CISS scores for students without amblyopia or strabismus remain (unexpectedly) high.

Exploring whether there is a physiological basis for the high symptom scores, as distinct from uncorrected refractive error, has not been explored in depth here, and hence remains to be investigated under more rigorous conditions than a typical screening. Although we suspect that unidentified binocular problems are a likely cause [41, 42, 50], few studies to date have looked at the true prevalence of oculomotor or binocular vision anomalies in large cohorts of unselected school children [78]. Previous studies indicate that based on the criterion of a single binocular vision sign, as many as 39% of children appear to have at least one aspect of impaired functional vision [60] whereas based on multiple criteria it is likely that at least 10% of school children in Australia [60] the USA [41, 79] and Sweden [17] will have a significant accommodative or convergence problem, or both. Currently, literature investigating the reading distance adopted for reading/writing at a desk or during near leisure activities [80], especially for children [44, 81], is scarce. In addition, the emerging worldwide use of handheld devices and associated short reading distances is concerning. For example, one study [81] of fully-corrected myopic Chinese 6 to 13 year old children found the viewing distance was 21.3 ± 5.2 cm whilst playing handheld video games and 24.9 ± 5.8 cm whilst writing. This is especially concerning given that video games on handheld devices are often played for durations greater than 1 h [82]. With adults, it has also been noted that the viewing distance when using a smartphone decreases significantly and the degree of asthenopia increases over a 1 h viewing period [83]. The impact upon symptomatology of factors such as screen time, lighting, font size, distance from the device, etc., do not appear to have been explored in depth in children in the new era of digital media and merit investigation. Unfortunately, two recent studies on asthenopia during use of handheld devices both excluded those with amblyopia and/or strabismus [44, 83].

### Educational value

The pursuit of ophthalmic drivers behind asthenopic symptoms associated with near work in in children should be an important issue given that ocular discomfort has been shown to act as a disincentive to reading and academic progress [37, 45–52]. The clinical guidelines from the professional association of ophthalmologists in the USA recommends prescribing for children with asthenopia [84]. Therefore, the CISS could become a clinically valuable and useful tool to facilitate decision-making related to prescribing for refractive error, though worryingly, some ophthalmic practitioners do not subscribe to prescribing on the basis of the presence of symptoms alone [77, 85]. Evidence exists indicating that uncorrected hyperopia during school years is more commonly linked to impaired cognitive functions [86], impaired literacy [51] and impaired visual motor integration [87]. The current study showed moderate to high hyperopes to be highly symptomatic, suggesting that they may be more likely to benefit from refractive correction and thus minimize educational disadvantage. Correction of visual anomalies with spectacles in middle school children has also been found to lead to reporting of less symptoms [65], though for not all children, as even some emmetropic children diagnosed with reading and writing difficulties still report significant asthenopic symptoms [45]. Either way, our study suggests that a more comprehensive visual assessment than simply refractive error is required if best quality of life and academic outcomes are sought.

## Conclusion

Our general school screening has demonstrated that nearly half the students have visual complaints, a proportion well above that found overseas. Although this asthenopia in middle school students was not related to refractive errors per se, our highest CISS scores were related to higher hyperopia and strabismus and amblyopia. Thus, our findings suggest that future studies should investigate relationships between asthenopia and prolonged accommodative-convergence effort at near, especially given the increasingly more frequent daily use of computers and handheld devices in cognitively challenging and leisure situations.

## Data Availability

The datasets used and/or analysed during the current study are available from the corresponding author on reasonable request.

## Abbreviations

CI: Convergence insufficiency
CISS: Convergence Insufficiency Symptoms Survey
CITT: Convergence Insufficiency Treatment Trial
ETDRS: Early Treatment Diabetic Retinopathy Study
RESC: Refractive Error Study in Children
SER: Spherical equivalent refraction
